# Bibliometric analysis of the links between embryo transfer and endometrial receptivity: Mapping knowledge landscapes and emerging trends (2005–2024)

**DOI:** 10.1097/MD.0000000000042014

**Published:** 2025-04-25

**Authors:** Chen Xu, Chuanhui Zhang, Shu Xu, Jingli Ma, Lingling Ran

**Affiliations:** a Department of Obstetrics and Gynecology, Chaoyang Central Hospital, Chaoyang City, Liaoning Province, China; b Department of Joint and Sports Medicine, Chaoyang Central Hospital, Chaoyang City, Liaoning Province, China.

**Keywords:** bibliometrics, Bibliometrics, Citespace, embryo transfer, endometrial receptivity, VOSviewer

## Abstract

**Background:**

Infertility is a significant global reproductive health challenge with varying prevalence across regions. While assisted reproductive technologies, especially in vitro fertilization, have created new possibilities for treating infertility, embryo transfer success remains limited by multiple factors, particularly endometrial receptivity. Understanding the relationship between embryo transfer and endometrial receptivity is crucial for advancing reproductive medicine and improving infertility treatment outcomes. To address the lack of bibliometric analysis in this field, we conducted a comprehensive bibliometric study of relevant academic literature.

**Methods:**

We analyzed articles and reviews on endometrial receptivity and embryo transfer from the Web of Science Core Collection using CiteSpace, VOSviewer, and Bibliometrix package. The analysis focused on countries, institutions, journals, authors, keywords, and references.

**Results:**

We identified 1478 documents published over 2 decades, showing an upward trend in annual publications. China led in publication volume, while the USA had the highest citation impact. The University of Valencia and Shanghai Jiao Tong University were the most productive institutions, with fertility and sterility being the leading journal. Simón, C emerged as the most influential author based on publication count and citations. Key research areas included comparing fresh versus frozen embryo transfer, addressing repeated implantation failure, and improving endometrial receptivity. The endometrial receptivity array for personalized embryo transfer represents an emerging research direction in reproductive medicine.

**Conclusion:**

This first comprehensive bibliometric analysis of embryo transfer and endometrial receptivity research provides valuable insights into knowledge development, research hotspots, and future directions in the field, serving as a crucial reference for scholars in reproductive medicine.

## 1. Introduction

In recent years, infertility has emerged as one of the globally significant health concerns, with an increasing prevalence year by year. In China, the incidence of infertility has risen from 11.9% in 2007 to 17.6% in 2020,^[1]^ highlighting the gravity of the issue. The diversity of factors contributing to this upward trend could include lifestyle changes, environmental pollution, a rise in reproductive diseases, and societal pressures.^[2]^

In the backdrop of escalating infertility rates, the application of in vitro fertilization and embryo transfer (IVF-ET), holds a significant societal and medical value.^[3]^ IVF-ET technology refers to taking eggs collected from a woman’s ovaries and fertilizing them with sperm in vitro. The fertilized eggs are then continuously cultured until they reach the cleavage-stage embryo or blastocyst stage. During the implantation window of the endometrium, the cleavage-stage embryo or blastocyst will be transplanted into the uterine cavity, where it implants and develops into a fetus.^[4]^ Consequently, successful implantation necessitates a productive crosstalk among the endometrial lining and the blastocyst.^[5,6]^ The capacity of the endometrium to permit the blastocyst to localize, adhere, penetrate, implant and facilitate the development of the embryo can be regarded as endometrial receptivity.^[7]^ It constitutes one of the crucial elements for successful embryo implantation and serves as an important connection in determining the success of assisted reproductive technologies (ART). Hence, it is necessary to investigate the influence of endometrial receptivity on embryo transfer, so as to provide more accurate and effective intervention and treatment guidance and thereby increase clinical embryo implantation success rates.

Nevertheless, there is a dearth of scientometric methodologies employed to quantify the analysis of information and data pertaining to endometrial receptivity and embryo transfer, thereby providing a scientific foundation and decision-support framework. So, the objective of this manuscript is to address the aforementioned gap in the literature through a comprehensive bibliometric analysis. For this study, multiple software programs were utilized for analyzing related literature on endometrial receptivity and embryo implantation and for constructing scientific knowledge maps. This study has 4 main research objectives: (Ⅰ) recognize the principal producers in the field of embryo transfer and endometrial receptivity from 2005 to 2024, encompassing nations, institutions, journals, and authors; (Ⅱ) probe into the shift of research priorities; (Ⅲ) predict the forthcoming research horizons of this Domain; (Ⅳ) call for increased attention to this subject matter from professionals, particularly clinicians, and researchers. This bibliometric analysis provides a thorough examination of the knowledge repository, research focal points, and potential future directions in this domain. It may act as a crucial reference for scholars in the relevant field.

## 2. Materials and methods

### 2.1. Data supply

The Web of Science Core Collection (WoSCC), provided by Clarivate Analytics based in Philadelphia, PA, stands as one of the foremost authoritative citation repositories within the domain of bibliometrics.^[8]^ With comprehensive data collection and powerful indexing features, it contains basic information on the article’s country, organization, and author keywords, and is particularly noteworthy for its detailed bibliographic information. Thus, this database is considered highly suitable for bibliometric analysis and is widely utilized. For this study, we selected publications related to embryo transfer and endometrial receptivity within the WoSCC database, specifically in the Science Citation Index Expanded.

### 2.2. Data retrieval strategy

**Figure 1.**
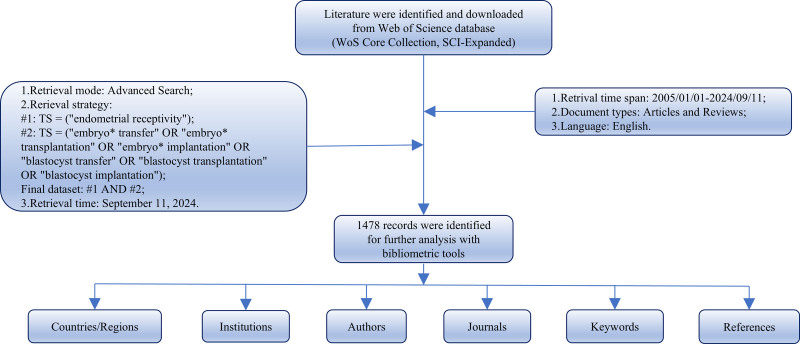
Study flow diagram.

In light of the database may be updated each day as it is still operational, 2 authors carried out an extensive online search within a span of 1 day to avoid deviations. When differences of opinion are encountered, the search strategy is adjusted and the team reaches agreement after a comprehensive evaluation. Comprehensive collection of all possibly relevant publications is accounted for according to the topic (TS) with the following search formula: [#1: TS = (“endometrial receptivity”); #2: TS = (“embryo* transfer” OR “embryo* transplantation” OR “embryo* implantation” OR “blastocyst transfer” OR “blastocyst transplantation” OR “blastocyst implantation”); Final dataset: #1 AND #2]. The core use of wildcards (*) is to match or replace one or more characters, which we can use to enhance the comprehensiveness of the search. For example, embryo* would also replace the terms of embryos, embryonic, and so on. The period was then set to 20 years, and only studies published between 2005 and 2024 were encompassed. English was the only language to which the language of literature was limited. English was the sole language used in the literary context, with the scope of literature confined to articles and reviews, adhering to certain exclusion criteria depicted in Fig. 1. In order to avoid data bias resulting from database updates, the literature search was accomplished on September 11, 2024. Eventually, 1478 publications were encompassed. The “Full Record and Cited References” of all searched documents were downloaded and exported in tab-delimited (UTF-8) format or plain text to facilitate bibliometric analysis. To ensure the reproducibility and transparency of the research, we have provided the complete raw data as supplementary materials (Data S1**–**S3, Supplemental Digital Content, https://links.lww.com/MD/O737; https://links.lww.com/MD/O738; https://links.lww.com/MD/O739).

### 2.3. Data analysis

Bibliometrics constitutes the scholarly exploration of the quantitative facets of literature, encompassing the analysis of citation patterns, publication trends, and network structures within academic domains.^[9,10]^ In our study, bibliometric visualization was carried out using CiteSpace 6.3.R1, VOSviewer 1.6.20, and Bibliometrix 4.4.1 (R-Tool of R-Studio). Concurrently, the Boltzmann curve in OriginPro 2024 has been employed to model and analyze the growth trend of annual publications in this research domain. All the primary data utilized in this research were retrieved from public databases. Consequently, there was no need for ethical review.

In the realm of bibliometric analysis, CiteSpace, a JAVA-based software tool, stands out as a pivotal tool, and it has garnered widespread adoption across the scholarly community.^[11]^ It adeptly identifies and elucidates the contributions of nations and institutions, mapping out the intricate web of scholarly collaborations. Furthermore, it dissects the disciplinary landscape, shedding light on the distribution of research efforts across various fields. By quantifying citations and co-citations, CiteSpace unveils the prominence and interconnectivity of research works, thereby pinpointing areas of intense scholarly interest, namely research hotspots, which are indicative of the pulse of current scientific inquiry. This multifaceted capability renders CiteSpace an indispensable asset in the arsenal of researchers and analysts alike, facilitating a nuanced understanding of the scholarly ecosystem.^[12]^

VOSviewer, a bibliometric analysis tool, which is recognized for its capabilities in visualizing scientific literature networks.^[13]^ VOSviewer facilitates the generation of visual network representations, delineating collaborative relationships, and keyword co-occurrence patterns within the scientific literature.^[14]^ VOSviewer is designed with the primary objective of offering users a comprehensive perception of the dynamics and structure of scientific research.

Bibliometrix is a robust software package reliant on the R programming language, frequently utilized for bibliometric analysis, encompassing data importation, format transformation, data cleansing and organization, descriptive statistics, co-occurrence matrix construction, data normalization, and mapping visualization.^[15]^

## 3. Result

### 3.1. Trends of annual publications and citations

In line with the previously outlined search methodologies, we have extracted a comprehensive data set comprising 1478 scholarly articles from WoSCC database for the purpose of our analytical inquiry. These publications have a total citation count of 41,201 (32,887 without self-citations). This equates to a mean citation frequency of 27.88 per document. The entire corpus has an H-index of 91, which is a reliable metric for measuring scientific impact.

**Figure 2.**
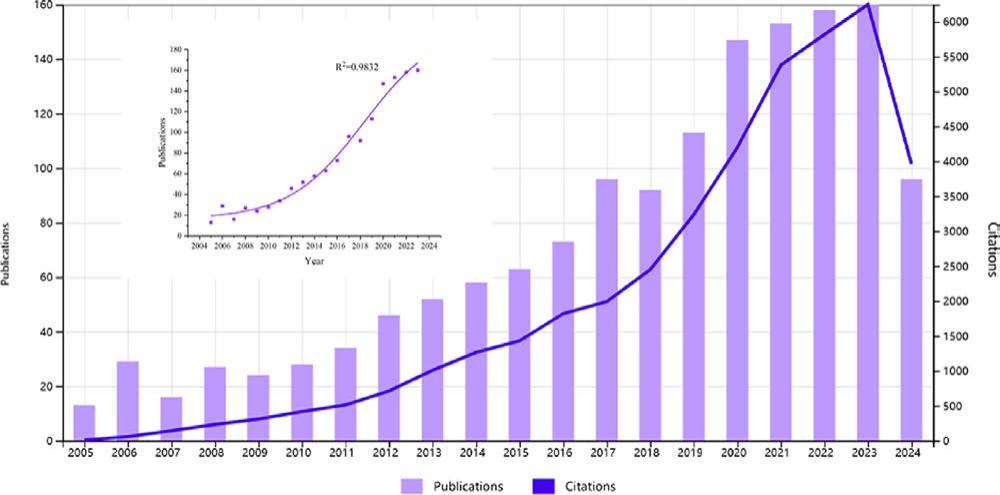
Trends in the literature related to embryo transfer and endometrial receptivity over the past 2 decades.

Fig. 2 shows the number of annual publications, citation frequency and trends, and a fitted curve of annual publications (*R*^2^ = 0.9832) from 2005 to 2024 (with 2024 data only up to September 11). There is no doubt that the trend in both publication output and citation frequency over the 2-decade period under review is upward. The annual growth rate for articles was 11.73%. The year 2023 was the peak of publication activity within this time frame, with a total of 160 articles documented as of September 11, 2024.

Moreover, the line graph displayed alongside Figure 2 demonstrates the secular trend in the annual average citation rate for the articles pertinent to our subject matter, spanning the period from 2005 to 2024 (the specific annual publication volume is available in the Data S4, Supplemental Digital Content, https://links.lww.com/MD/O740). It can be observed that the annual citation rate has maintained an unbroken pattern of ascent, a phenomenon that persists irrespective of fluctuations in the annual publication volume. It is worthy of note that a significant rise in the number of citations occurred between 2020 and 2023, reaching a peak of 6246 in 2023. This surge in citations reflects a growing interest in and influence of research in this domain.

### 3.2. Country/region analysis

**Figure 3.**
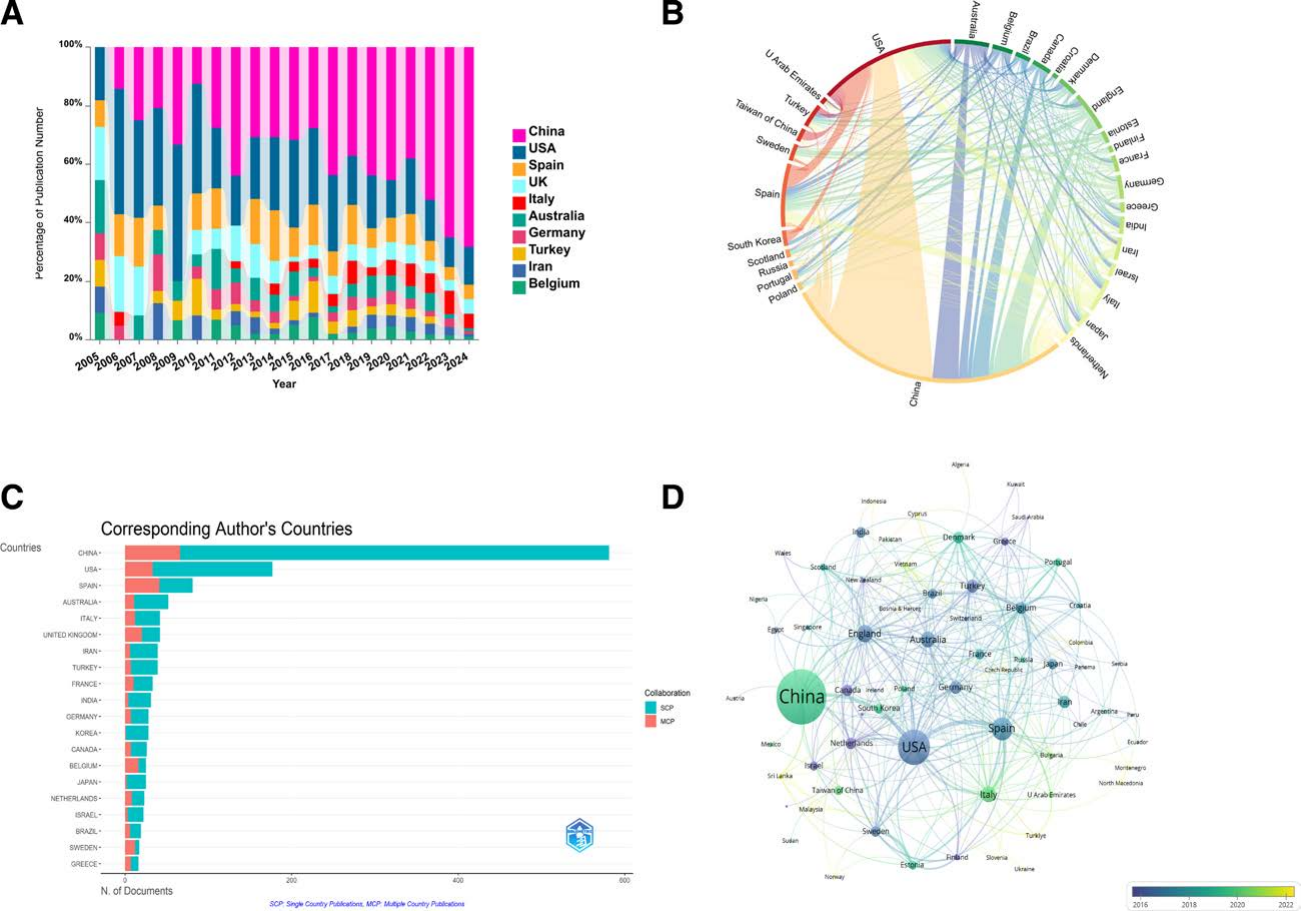
Contribution analysis of countries/regions in research. (A) The evolving share of annual scholarly outputs among the top 10 publishing nations from 2005 to 2024. (B) Linkages between the top 30 countries/areas leading regarding the count of publications. (C) Cooperative efforts among countries/regions with corresponding authors. (D) Analysis of country co-authorship using VOSviewer. On this interactive visualization map, each node signifies a country, while the connections among them depict collaborative authorships. he dimensions of each node scale according to the aggregate publication count. The color of each node reflects its corresponding average year of publication (AAP), as indicated by the color gradient displayed in the bottom right corner.

Publications encompassed 70 countries/regions (the specific data can be found in Data S5, Supplemental Digital Content, https://links.lww.com/MD/O741). China published the most documents, with (567, 38.36%), followed by the United States (264, 17.86%), Spain (122, 8.25%), England (77, 5.21%), and Australia (67, 4.53%). China and USA collectively account for 51.29% of the global publication output, significantly surpassing contributions from other nations. Figure 3A shows the evolution of annual publication trends in the ten countries or regions that have contributed most to this field of study. The figure shows that China’s annual publications, which were initially low, have since shown strong growth, and overtaking the USA regarding the quantity of articles issued in 2011. Figure 3B clearly shows international cooperation between countries and regions. The percentage of arcs reflects the amounts of articles published, and the width of the connecting streaks indicates the intensity of collaboration. The figure demonstrates that China and the USA cooperate closely, while the USA and Spain have a more intense partnership. Figure 3C clearly shows that although China and the USA ranked first and second, respectively. Regarding the quantity of published articles, most of these results were the result of collaboration between domestic researchers. This indicates that there are certain limitations in international academic exchange and cooperation between the 2 countries. In contrast, Sweden (12 papers, 70.6%), Belgium (16 papers, 64.0%), Spain (41 papers, 50.6%), the United Kingdom (20 papers, 47.6%), and Greece (7 papers, 43.8%) are far ahead in terms of international collaborations, occupying the 5 leading locations with regard to the quantity of multinational publications (MCPs) compared to all documents in their own countries (MCP ratio). This is clear proof of their unquestionable active participation and openness to global research collaboration on a global scale. Although China holds the highest count of multinational publications (66), it is not situated among the leaders 5 with regard to the multinational co-authorship publication (MCP) ratio due to its having published the largest number of documents. The USA ranks second in terms of total article output, yet it has a relatively a small quantity of collaborative publications with other countries (33), hence its low percentage of multilateral partners (Table 1). International cooperation between different countries/regions is also visualized in Figure 3D generated by VOSviewer, where the linking lines among them signify the cooperation endeavors. The thickness of the lines indicates the strength of the cooperation. The countries/regions are color-coded in accordance with their average year of occurrence (AAY). Blue denotes earlier participation in the area, whereas yellow represents later involvement.

**Table 1:** Top 10 countries in terms of number of publications, frequency of citations, and total associations intensity.

Rank	Countries	Documents	Countries	Total Citations	Countries	Total link Strength
1	China	567	USA	11,793	China	4610
2	USA	264	China	8973	USA	4342
3	Spain	122	Spain	5601	Spain	3492
4	England	77	England	3380	England	1556
5	Australia	67	Australia	2899	Australia	1287
6	Italy	67	Israel	2539	Italy	1172
7	Germany	46	Netherlands	2015	Belgium	1127
8	Turkey	46	Italy	1948	Israel	914
9	Iran	44	Germany	1889	Sweden	903
10	Belgium	567	Belgium	1680	Denmark	829

### 3.3. Analysis of the most prolific institutions

**Figure 4.**
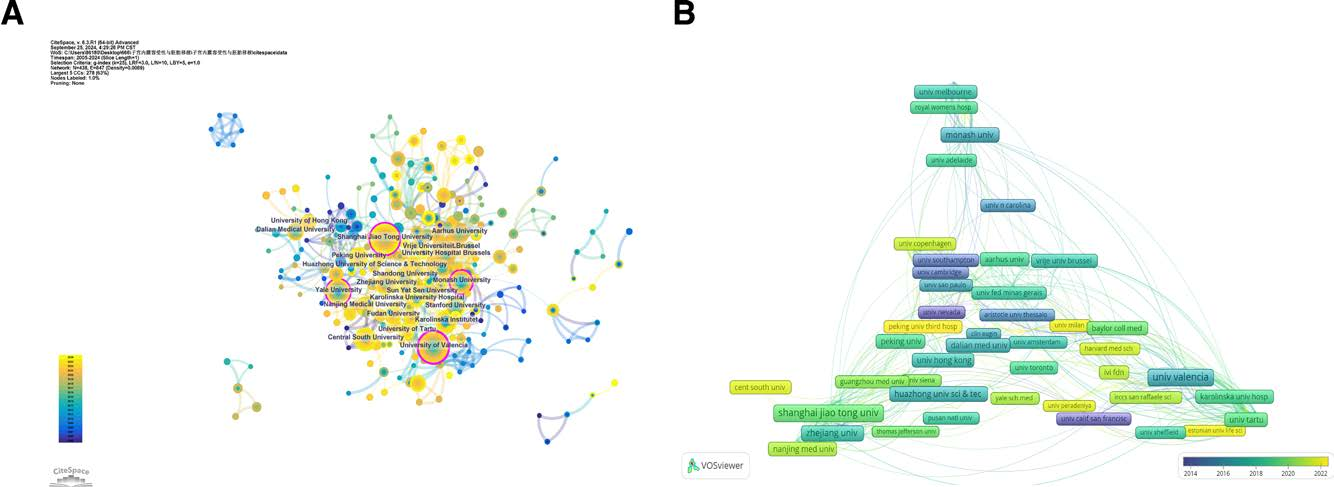
Examination of research-affiliated institutions. (A) Institutional collaboration investigation by CiteSpace. On this network diagram, each node signifies an institution, with its size indicative of the relative volume of research outputs. A node encircled in purple indicated a centrality value exceeding 0.1. (B) VOSviewer-based analysis of institutional co-authorship networks.

CiteSpace was used to carry out institutional collaboration analysis (**Fig. 4A**). The size of the nodes is in proportion to the quantity of papers published. The quintet of institutions with the most substantial research output, as evidenced by paper contributions, are the University of Valencia (55 publications), Shanghai Jiao Tong University (55 publications), Zhejiang University (39 publications), Fudan University (28 publications), and Huazhong University of Science and Technology (27 publications), which occupy distinguished positions within the network analysis (data are detailed in Data S6, Supplemental Digital Content, https://links.lww.com/MD/O742). Notably, certain nodes in the graph are encircled with a purple halo, highlighting the high betweenness centrality (BC > 0.1) of these institutions within the network. Centrality is a key metric for measuring the importance and impact of a node within the meshwork; nodes with high centrality often have the power to influence and regulate the information stream and the distribution of resources within the network. The University of Valencia, Shanghai Jiao Tong University, Monash University, and Yale University, with their high centrality, have become de facto “bridging institutions.” They not only have strong research capabilities but also promote the optimization and allocation of global scientific resources and the acceleration of scientific innovation through extensive cooperation and exchange. Figure 4B, generated by VOSviewer, is an institutional co-authorship network graph where nodes representing institutions are color-categorized in accordance with their average age of publication (AAY). As indicated by the color gradation in the bottom right quadrant, certain institutions, including the University of Nevada and the University of California, San Francisco, are highlighted in purple, signifying a lower average attention year (AAY). This suggests that these institutions’ researchers are among the pioneers in the field. Conversely, those marked in yellow are indicative of more recent entrants to the research domain.

### 3.4. Analysis of influential authors

**Figure 5.**
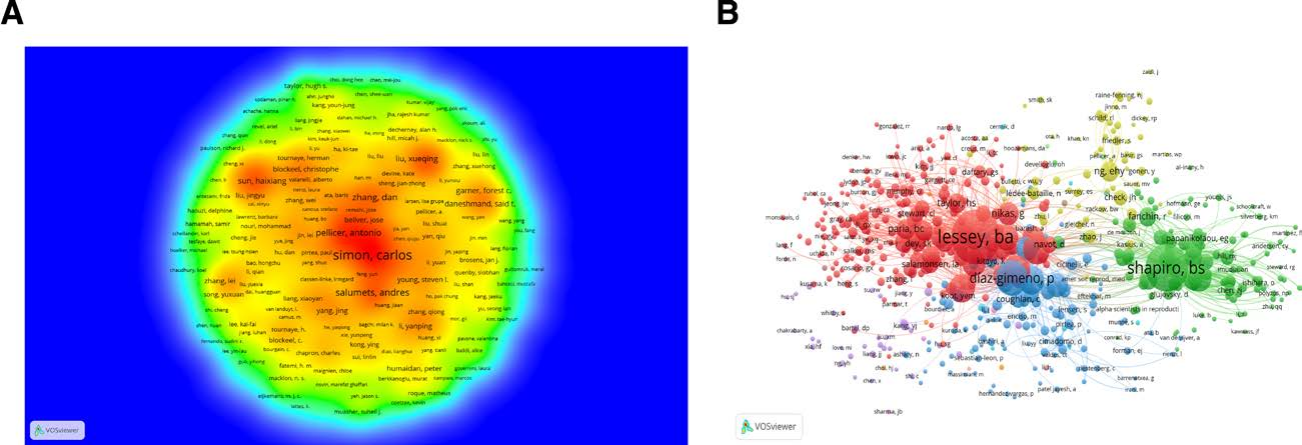
Visualization of association graphs of coauthors and co-cited authors in endometrial receptivity and embryo transfer. (A) VOSviewer-facilitated analysis of author co-authorship relationships. On the author co-occurrence density map generated by VOSviewer, the dimensions of each node reflect the prominence or weight of the respective authors, whereas the node’s hue serves as an indicator of the frequency of their collaborative occurrences. As the color becomes warmer, it signifies a greater density of co-occurrence, indicating stronger collaboration among authors and a higher concentration of research endeavors. (B) Co-cited author analysis by VOSviewer. Nodes sharing the same color denote they belong to the same cluster, whereas nodes of varying hues represent authors engaged in distinct collaborative relationships. The size of the words, the diameter of the circles, and the thickness of the connections all scale directly with the frequency of co-citation.

In the realm of scientometrics, the frequency of a researcher’s publication output is often considered a metric for gauging the intensity of research activity and the extent of scholarly contributions within a specific academic domain. This study has identified a corpus of 1478 scientific papers, which represent the academic contributions of over 6000 authors. Figure 5A illustrates a co-occurrence network density plot based on author collaboration, incorporating only those authors who have published a minimum of 5 papers (Data are detailed in Data S7, Supplemental Digital Content, https://links.lww.com/MD/O743). Regarding publication frequency, Simon, Carlos, from the University of Valencia, Spain, leads the list with the highest publication count, succeeded by Salumets, Andres, originating from the University of Tartu, Estonia. Tied for the third position are Pellicer, Antonio, from the University of Valencia, Spain, Salamonsen, Lois A, affiliated with the University of Melbourne, Australia, and Zhang, Dan, affiliated with Zhejiang University, China.

The concept of co-citation relationships is defined by the occurrence of at least one instance where 2 authors are collectively referenced within the bibliographic citations of a third-party scholarly work. Co-citation analysis of authors serves as a commonly employed methodology for uncovering the pivotal nodes within the co-citation web of a particular research domain. Significant academic impact within their domain tends to be exerted by authors who are often co-cited, whereas those who are often jointly cited may share thematic similarities or interdisciplinary research interests. Utilizing VOSviewer software, an in-depth examination of the author co-citation meshwork was conducted, identifying 575 authors with more than 20 citations each (Fig. 5B). Based on the diverse research directions and domains of the authors, the analysis has delineated 5 distinct clusters, each represented by a unique color. Among these authors, top 3 placement with the highest Total Link Strength (TLS) are Salamonsen, LA, from Monash University, Australia (18,964), Shapiro, BS, from the Las Vegas Fertility Medical Center, USA (12,470), and Diaz-Gimeno, P, from the Instituto Valenciano de Infertilidad, Spain (9879). Table 2 lists the leading 10 authors and cited authors in the field.

**Table 2:** Top 10 authors and cited authors.

Rank	Author	Documents	Countries	Institution	Author	Citations	Countries	Institution
1	Simon, Carlos	31	Spain	University of Valencia	Lessey, Ba	624	USA	Atrium Hlth Wake Forest Baptist
2	Salumets, Andres	18	Sweden	Karolinska Institutet	Shapiro, Bs	510	USA	University of Nevada Las Vega
3	Pellicer, Antonio	17	Spain	University of Valencia	Díaz- gimeno, P	337	Spain	Instituto de Investigación Sanitaria La Fe
4	Salamonsen, Lois A.	17	USA	Hudson Institute of Medical Research	Horcajadas, J. A.	266	Spain	Universidad Pablo de Olavide
5	Zhang, Dan	17	China	Zhejiang University	Haouzi, D	265	France	Universite de Montpellier
6	Ruiz-alonso, Maria	16	Spain	R&D Paterna Valencia	Maria Ruiz-Alonso	256	Spain	R&D Paterna Valencia
7	Kuang, Yanping	13	China	Shanghai Jiao Tong University	Altmäe, S	248	Spain	Karolinska University Hospital
8	Liu, Xueqing	13	Belgium	Ghent University	Achache, H	228	Israel	Hebrew University of Jerusalem
9	Sun, Haixiang	13	China	Nanjing University	Roque, M	225	France	Universite de Bordeaux
10	Shapiro, Bruce S.	12	USA	University of Nevada Las Vegas	Noyes, Rw	218	USA	Smithsonian Institution

### 3.5. Analysis of the higher-impact journals

**Figure 6.**
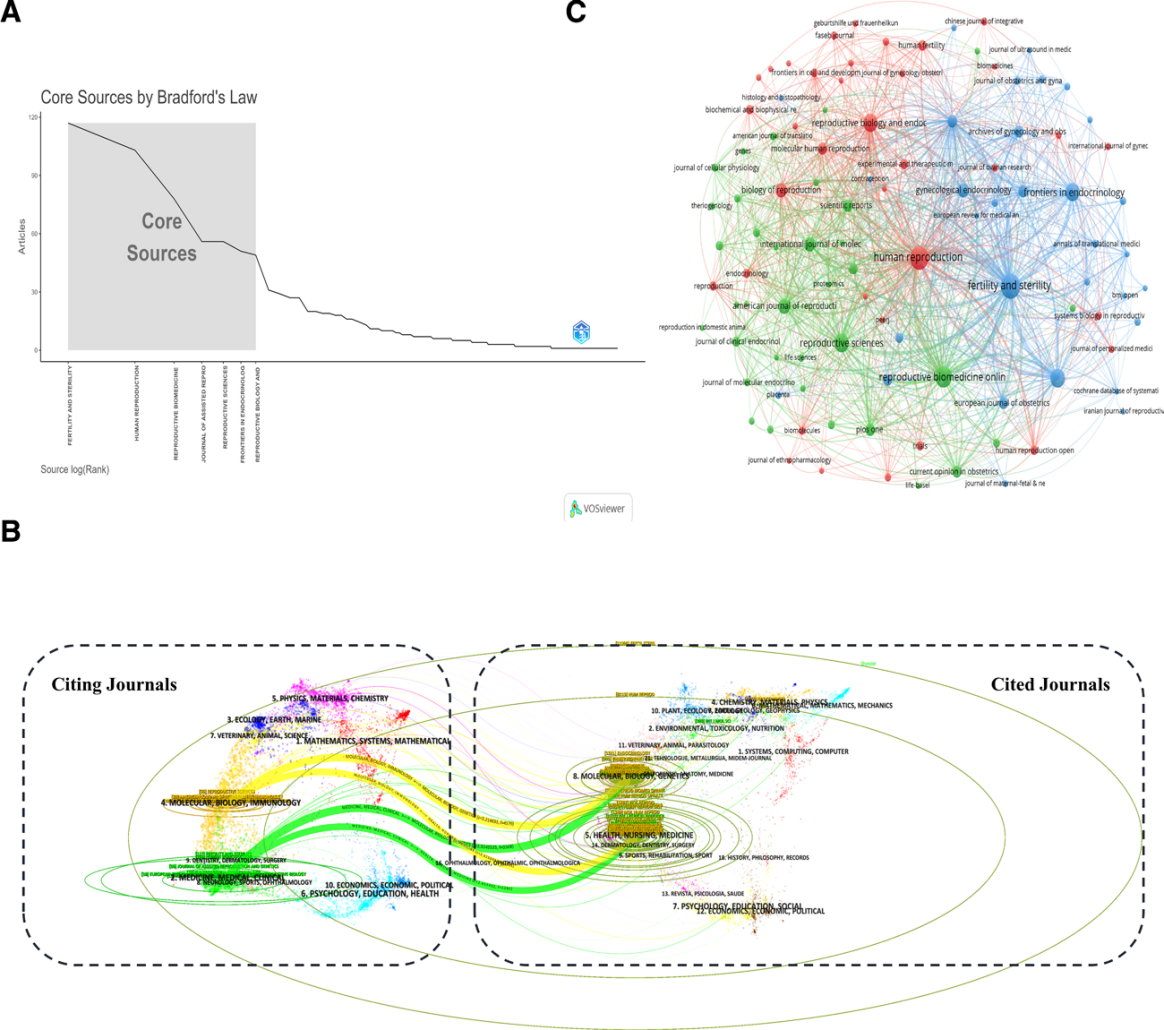
An analysis of scholarly journals pertaining to endometrial receptivity and embryo transfer. (A) An application of Bradford Law to academic journals. (B) A superimposed dual-map visualization of journals focusing on endometrial receptivity and embryo transfer research. On the left are clusters of citing journals, while on the right are the cited journals. The colored trails connecting them signify the cited relationships between them. (C) A VOSviewer-generated network visualization map depicts the analysis of journal co-citations. The dimensions of the nodes are proportional to the volume of citations they receive.

In accordance with Bradford Law, we have identified 7 core journals, as depicted in Figure 6A, which including *Fertility and Sterility*, *Human Reproduction*, *Reproductive BioMedicine Online*, *Journal of Assisted Reproduction and Genetics*, *Reproductive Sciences*, *Frontiers in Endocrinology*, and *Reproductive Biology and Endocrinology*. These journals have a significant publication volume in the relevant fields, are recognized for their high-quality content, and exert substantial influence, thus constituting the core periodicals within this academic discipline (Data S8, Supplemental Digital Content, https://links.lww.com/MD/O744 shows the most relevant sources). Figure 6B depicts a journal bipartite graph overlay map, visually depicting the spread of scholarly periodicals, along with the path of citation patterns, and the trajectory in citation trends. In the journal bipartite graph overlay map, the left-hand side denotes journals that act as citations, whereas the right-hand side denotes those that are cited. The color-coded lines interlink journals in the left part with those in the right part, delineating the directionality and contextual framework of citation relationships. Upon examination of the bipartite graph overlay of journal publications, we have identified 4 primary citation pathways (highlighted in yellow and green), with citing journals predominantly falling within the realms of biology, immunology, clinical medicine, and other research disciplines. On the other hand, the cited journals exhibit a broad distribution across various fields, including molecular biology, genomics, obstetrics, environmental science, nutrition, nursing, pharmacy, zoology, and other areas of study.

### 3.6. Visual exploration of keywords

#### 3.6.1. Analysis of keyword co-occurrence

**Figure 7.**
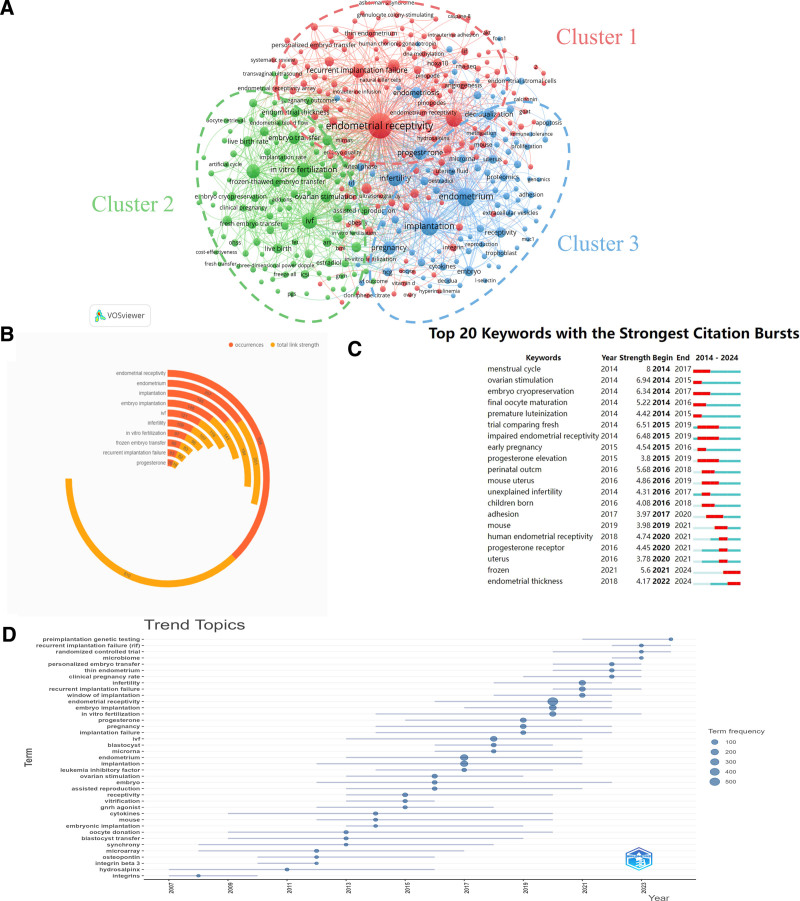
Analysis of keywords and research trends related to the research. (A) Visual graph of keyword co-occurrence network analysis. On this network map, keywords that exhibit close associations are grouped into clusters distinguished by a uniform color. All the keywords can be categorized into 3 distinct clusters: cluster 1 (red nodes), cluster 2 (green nodes), cluster 3 (blue nodes). (B) Frequency distribution of the top 10 most frequent occurrences keywords and their total link strength. (C) The top 50 keywords exhibiting the most significant citation bursts, as identified by CiteSpace. A blue line depicts the timeline, whereas the red bars signify burst periods, encompassing the commencement year, conclusion year, and the duration of the keyword’s burst activity. (D) Trend topics analysis.

Keywords serve as a concentrated distillation of the essence of a scholarly article, reflecting the core themes, subjects, methodologies, and conclusions of the research presented. They can be effectively utilized to dissect the foundational underpinnings, prevailing focal points, and cutting-edge advancements in the domain of embryo transfer and endometrial receptivity. Within the scope of this research, a keyword co-occurrence meshwork map was built utilizing the VOSviewer software. Given the variability in the forms of keywords assigned by multiple authors, yet their equivalence in meaning, a manual consolidation was performed. From a corpus of 1478 publications, a total of 2505 author-assigned keywords were extracted. By setting a minimum co-occurrence threshold of 3, a network comprising 350 nodes was generated, as depicted in Figure 7A, which illustrates the superimposed visualization map. Examination of the network diagram reveals a robust co-occurrence relationship throughout the cluster analysis. Numerous nodes are present, which, based on the distinct attributes pertaining to of the keywords, can be broadly categorized into 3 distinct clusters.

Red cluster (Cluster 1): related to reproductive health and disease. Keywords such as endometrial receptivity, recurrent implantation failure (RIF), hydrosalpinx, and Asherman syndrome. This cluster predominantly focuses on diseases and conditions that impact reproductive health, such as infertility, implantation failure, hydrosalpinx, and endometrial adhesions. The research is centered on the diagnosis and treatment of these pathologies, as well as their implications for reproductive health and the success rates of pregnancy.

Green cluster (Cluster 2): related to reproductive technology and clinical practice. Keywords for example: IVF, embryo transfer, in vitro fertilization, ovarian stimulation. This cluster is dedicated to the field of reproductive technology, encompassing IVF, embryo cryopreservation, oocyte retrieval, and ovarian stimulation, among others. These keywords pertain to the clinical practices and therapeutic outcomes of the reproductive process. The research is concentrated on enhancing the efficacy and success rates of reproductive technologies, such as optimizing techniques for embryo cryopreservation and transfer, and refining protocols for ovarian stimulation.

Blue clustering (Cluster 3): related to reproductive physiology and molecular mechanisms. Keywords include implantation, endometrium, progesterone, adhesion, and apoptosis. This cluster focuses on reproductive physiology and molecular mechanisms, particularly implantation, embryo quality, gene expression regulation, proteomics, and cellular signal transduction. Research within this domain can explore the molecular mechanisms affecting embryo implantation and endometrial receptivity, utilizing genomic and proteomic approaches to identify novel biomarkers.

#### 3.6.2. Keyword high-frequency analysis

Figure 7B shows the distribution of the top 10 most frequent keywords and their total link strength. It can be noted that, aside from the search term, other predominant keywords within this research field comprise endometrium, implantation, IVF, and infertility. Keywords with high occurrence frequency and high total link strength are likely to represent core issues or research hotspots within the field, which are of significant importance for understanding the knowledge structure and research trends within the domain.

#### 3.6.3. Keyword burst analysis

As illustrated in Figure 7C, we utilized the CiteSpace software to identify 20 keywords that have experienced a surge in frequency. Notable keywords characterized by a sustained burst include trial comparing fresh (from 2015–2019), impaired endometrial receptivity (from 2015–2019), and progesterone elevation (from 2015–2019). The keywords with the highest burst intensity are, in order, menstrual cycle (8), ovarian stimulation (6.94), and a trial comparing fresh (6.51).

#### 3.6.4. Keyword evolution analysis

Furthermore, a Trend Topics study was performed utilizing the bibliometrix package to perform a time series analysis of keywords in the endometrial receptivity and embryo transfer dataset. This was done with the aim of identifying evolving research trends and topics over time. Figure 7D illustrates the evolution of the various themes over time by plotting the data. The lines on the graph represent the lifecycle of a topic, from emergence, through peak interest, to decline. It is notable that some topics have a relatively short span, indicated by a short line, indicating rapid fluctuations in interest, while others have a longer period of relevance. As depicted in the figure, foundational and core topics possess an extensive research history and sustained attention, such as hydrosalpinx, cytokines, embryo, and IVF. The current research trajectory is concentrated on directions like preimplantation genetic testing, RIF, and randomized controlled trials.

### 3.7. References and co-cited references

In the field regarding bibliometrics, citation investigation holds a central position. Although the value of citation rates is subject to some debate, the prevailing view is that the number of citations an article receives is indicative of its impact. Higher citation rates typically suggest greater academic value and guiding significance. Table 3 enumerates the 15 most highly cited scholarly articles, of which 8 were reviews and 7 were articles, with a significant proportion of the research published in medicine journals such as *Hum Reprod Update*, *Fertility and Sterility*, *Human Reproduction*, and *New England Journal of Medicine*. Specifically, an article published by Gellersen, B et al in *Endocrine Reviews*, cited 688 times, provides insight into the periodic alterations during the course of endometrial decidualization in the human endometrium in *Reproductive Health and Failure*.^[16]^ Decidual change, the mechanism through which endometrial stromal cells transform into secretory decidual cells, is a pivotal stage in embryo implantation and placentation. The regulatory mechanisms underlying this process are intricate, encompassing endocrine, paracrine, and autocrine factors, as well as the involvement of various transcription factors and signaling pathways. This decidualization process not only creates a nutrient-rich and immunologically privileged environment for the embryo but also exerts a significant influence in embryo selection and immune modulation through its dynamic changes. The second most-cited article is authored by Achache, H et al, and was published in *Human Reproduction Update* with 606 citations. This article discusses the role of endometrial tolerance markers in s prosperous embryo implantation.^[7]^ The article provides a comprehensive examination of various molecular markers, including adhesion molecules (such as integrins and cadherins), cytokines (such as leukemia inhibitory factor, LIF), mucins (such as MUC1), and prostaglandins, which exhibit significant expression changes during the window of uterine receptivity and are crucial for blastocyst adhesion and invasion. The publication by Chen ZJ *et al* in the *New England Journal of Medicine* ranks third in citation frequency (518). This article elucidates that in women with polycystic ovary syndrome undergoing infertility interventions, compared to fresh embryo transfer, frozen embryo transfer (FET) is correlated with an elevated incidence of live births and a diminished susceptibility to ovarian hyperstimulation syndrome, but may increase the risk of preeclampsia.^[17]^ In summary, the themes of the top 15 most-cited papers primarily encompass the physiological mechanisms of embryo implantation, a significant personalized embryo transfer (PET) through the endometrial receptivity analysis (ERA), a comparison between frozen-thawed and fresh embryo transfer, and RIF.

**Table 3:** Top 15 articles in terms of frequency of citation.

Rank	Title	Journal	First Author	Year	Cita- tions	DOI
1	Cyclic decidualization of the human endometrium in reproductive health and failure	Endocrine Reviews	Gellersen, B	2014	688	10.1210/er.2014-1045
2	Endometrial receptivity markers, the journey to successful embryo implantation	Human Reproduction Update	Achache, H	2006	606	10.1093/humupd/dml004
3	Fresh versus Frozen Embryos for Infertility in the Polycystic Ovary Syndrome	New England Journal of Medicine	Chen, ZJ	2016	518	10.1056/NEJMoa1513873
4	Evidence that the endometrial microbiota has an effect on implantation success or failure	American Journal of Obstetrics and Gynecology	Moreno, I	2016	480	10.1016/j.ajog.2016.09.075
5	Evidence of impaired endometrial receptivity after ovarian stimulation for in vitro fertilization: a prospective randomized trial comparing fresh and frozen-thawed embryo transfer in normal responders	Fertility and Sterility	Shapiro, BS	2011	450	10.1016/j.fertnstert.2011.05.050
6	Fresh embryo transfer versus frozen embryo transfer in in vitro fertilization cycles: a systematic review and meta-analysis	Fertility and Sterility	Roque, M	2013	400	10.1016/j.fertnstert.2012.09.003
7	Physiological and molecular determinants of embryo implantation	Molecular Aspects of Medicine	Zhang, S	2013	373	10.1016/j.mam.2012.12.011
8	Transfer of fresh versus frozen embryos in ovulatory women	New England Journal of Medicine	Shi, YH	2018	347	10.1056/NEJMoa1705334
9	Seminal plasma and male factor signaling in the female reproductive tract	Cell and Tissue Research	Robertson, SA	2005	343	10.1007/s00441-005-1127-3
10	The endometrial receptivity array for diagnosis and personalized embryo transfer as a treatment for patients with repeated implantation failure	Fertility and Sterility	Ruiz-Alonso, M	2013	336	10.1016/j.fertnstert.2013.05.004
11	Investigation and treatment of repeated implantation failure following IVF-ET	Human Reproduction	Margalioth, EJ	2006	330	10.1093/humrep/del305
12	Recurrent implantation failure-update overview on etiology, diagnosis, treatment and future directions	Reproductive Biology and Endocrinology	Bashiri, A	2018	318	10.1186/s12958-018-0414-2
13	Fresh versus elective frozen embryo transfer in IVF/ICSI cycles: a systematic review and meta-analysis of reproductive outcomes	Human Reproduction Update	Roque, M	2019	294	10.1093/humupd/dmy033
14	Conventional and modern markers of endometrial receptivity: a systematic review and meta-analysis	Human Reproduction Update	Craciunas, L	2019	274	10.1093/humupd/dmy044
15	Implantation failure: molecular mechanisms and clinical treatment	Human Reproduction Update	Cakmak, H	2011	256	10.1093/humupd/dmq037

**Figure 8.**
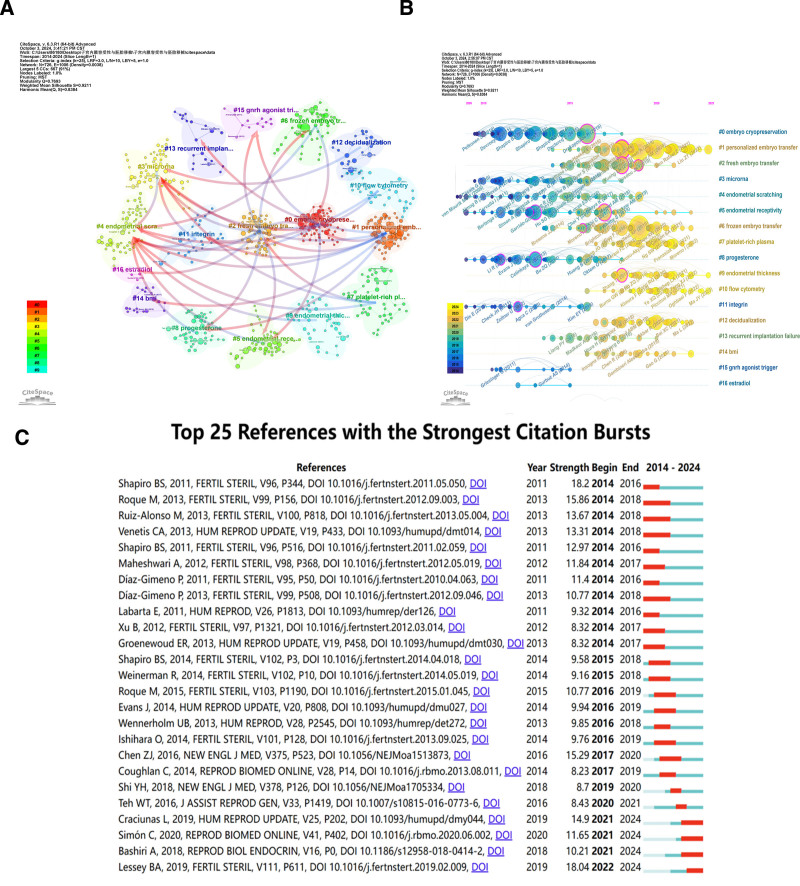
Analysis of references related to the research. (A) Keywords map of domain references. The keywords of the same color are in the same clusters, and the links represent the co-occurrence relationship between the keywords. The arrows indicate evolutionary relationships between clusters. The arrow points from one node to another, which indicates that the development of the former depends on the latter. (B) CiteSpace-facilitated co-citation analysis of references. On this timeline view map, the location of a node on the horizontal axis shows the time point of its first appearance. Lines that connect the nodes stand for co-cited relationships. The size of the node is in proportion to the citation count of the reference. A color closer to yellow indicates a time closer to 2023, and a bluer color means a time closer to 2000. (C) The top 20 references with the strongest citation bursts in the co-citation network.

A crucial feature of CiteSpace is the co-citation study of references, functioning as a tool for assessing the impact of scholarly works and elucidating the trajectory of disciplinary development. Frequently co-cited literature typically indicates research hotspots within a field, while widely co-cited references reflect the focal points of current investigations. Moreover, the recurrent co-citation of references suggests thematic similarities in content, and cluster analysis further reveals the common themes among these similar works, providing researchers with a nuanced perspective for understanding the structure and dynamics of their respective disciplines. By dynamically tracking co-citation relationships among literature, researchers are able to anticipate the developmental trends within academic disciplines. Particularly when emerging literature forms co-citations with traditional core documents, this may signify the birth of new research directions or the emergence of interdisciplinary fields. Based on the bibliometric evaluation performed in CiteSpace, Figure 8A illustrates the visualized network of cited references. It is worthy of note that the modularity value (Q value) and the average silhouette value (S-value) are 2 key indicators in terms of evaluating the significance of community architecture. Specifically, a Q value >0.3 typically signifies that the clustering structure is significant, while an S-value above 0.7 denotes a great degree of homogeneity and separation among the groups. In our investigation, the Q value stands at 0.7693, showing the rationality of the meshwork. The average silhouette value is 0.9211, signifying that these clusters exhibit great homogeneity. From the figure, it can be observed that “embryo cryopreservation” constitutes the largest cluster (#0), succeeded by “personalized embryo transfer” (#1) and “fresh embryo transfer” (#2). The “Cluster Dependencies” feature allows us to identify the interdependencies among various clusters. Multiple arrows from cluster #7, which pertains to platelet-rich plasma (PRP), point towards clusters #2 (fresh embryo transfer), #12 (decidualization), and #4 (endometrial scratching), indicating that the emergence of cluster #7 is contingent upon the development of these 3 clusters. Multiple arrows from cluster #7, associated with PRP, point towards clusters #2 (fresh embryo transfer), #12 (decidualization), and #4 (endometrial scratching), signifying that the emergence of cluster #7 is dependent on the evolution of these 3 clusters. Similarly, it can be inferred that cluster #4, endometrial scratching, as a foundational cluster, propels the development of various other groups, including #3 (microRNA), #8 (progesterone), #5 (endometrial receptivity), #9 (endometrial thickness), #7 (platelet-rich plasma), #6 (frozen embryo transfer), and #1 (personalized embryo transfer).

Simultaneously, we have presented a chronological perspective of the principal clusters in Figure 8B, enabling the rapid discernment of each cluster’s evolutionary traits as delineated by the timeline. It is evident that the research focus has undergone a shift from “embryo cryopreservation” (#0), “progesterone” (#8), “microRNA” (#3), “endometrial scratching” (#4), and “integrin” (#11) to “personalized embryo transfer” (#1), “frozen embryo transfer” (#6), “platelet-rich plasma” (#7), and “endometrial thickness” (#9). In the figure, we have also identified several highly central co-cited references with intermediary centrality, which often serve as pivotal bridges within the academic domain, facilitating the exchange and integration between different research directions or fields. Among them, the article with the highest intermediary centrality was authored by Tan J *et al.*, which explored the utilization of ERA in ART, marking a relatively novel area of research.^[18]^ The ERA technology, as a molecular gene diagnostic method, assesses the tolerance of the endometrium and the time of the window of implantation (WOI) through high-throughput sequencing and gene transcription profiling. This approach challenges the traditional notion that the WOI is a fixed period for all women. Based on the ERA test results, a personalized evaluation of individual endometrial receptivity can be achieved, thereby enhancing the success rate of embryo implantation. Figure 8C presents the top 25 references in this research field with the strongest citation bursts over the past 2 decades. It is evident that an article published by Shapiro BS in 2011 in *Fertility and Sterility* exhibits the highest intensity of citation bursts (18.2). Data S9, Supplemental Digital Content, https://links.lww.com/MD/O745 shows the top 20 most highly cited papers.

## 4. Discussion

### 4.1. Worldwide trends in the realm of embryo transfer and endometrial receptivity

Bibliometrics represents a methodological framework that utilizes quantitative analysis of literary data to outline the evolution, research priorities, and emerging trends in scientific and technological fields. By elucidating the structure and dynamics of research fields, bibliometrics provides researchers with valuable insights for rapidly appreciating academic information resources. Furthermore, it enables policymakers and decision-makers to grasp the progress of research, determine research hotspots, and prognosticate future research orientations. Additionally, it facilitates the evaluation of research project outcomes and enhances the efficient distribution of resources. In essence, Bibliometrics constitutes a powerful instrument that endows us with the capacity to deeply comprehend and navigate the intricate academic realm by uncovering patterns of knowledge production and interactions within the scholarly community.

In this study, a total of 1478 documents were included, comprising 1256 articles and 222 reviews. Regarding the trend in annual publication output, it can be demarcated into 3 distinct phases. From 2005 to 2010, this period can be regarded as the nascent phase of publication growth. Although the number of publications exhibited a gradual increase, the growth rate was relatively sluggish. This phase may represent the early development of the field or discipline, with researchers beginning to actively engage and disseminate their research findings. From 2011 to 2018, during this phase, a notable augmentation regarding the quantity of publications was observed. The purple line graph demonstrates a marked upward trend over this period, indicating that the volume of publications entered a period of rapid growth. This could be attributed to the field or discipline gradually maturing, attracting increased participation from researchers, or perhaps credit due to advancements in technology and methodology, which have enhanced research efficiency and output rates. From 2019 to 2024, although the volume of publications continues to exhibit a growth trend, the rate of increase may begin to decelerate during this phase, manifesting a state of stable growth. This suggests that the field has reached a relative maturity, with research directions and methodologies becoming progressively clarified and the themes and focus of research gradually stabilizing. Concurrently, this may also imply that competition within the field has intensified, necessitating a higher caliber of research outcomes to distinguish themselves amidst the plethora of literature. The annual citation counts have correspondingly manifested a similar upward tendency, and there is a positive correlation between them and the volume of published papers. This implies that the variation in citation trends can be ascribed to the rapid rise in the number of published documents.

In this field, China and the USA stand out as dominant forces among the principal contributors. They account for more than half of all publications and emerge as the leading contributors and drivers of research. However, it should be noted that certain nations, in spite of having a relatively lesser quantity of articles, display exceptionally elevated average citation rates per publication. Israel begins with a remarkable average citation rate of 104.20, followed by the Netherlands (68.2) and Estonia (54.3). This high citation rate is ascribed to the release of a number of extremely Impactful research reports. To illustrate, the article ranked second in citation frequency, authored by Achache, H et al from Israel and titled *Endometrial receptivity markers*, *The Journey To Successful Embryo Implantation* has garnered a total of 606 citations. The paper challenges the traditional histological assessment on the endometrium, suggesting that such assessment provides minimal clinically accessible information and advocating for the adoption of endometrial receptivity marker assessments.^[7]^ It offers a fresh viewpoint for comprehending the complex physiological process of embryo implantation. This seminal work was the first to delve into the connection among endometrial receptivity markers and successful embryo implantation, providing significant theoretical foundations and practical guidance for subsequent research.

From an institutional analytical standpoint, the University of Valencia, Shanghai Jiao Tong University, and Zhejiang University come to the fore as the top 3 institutions with regard to publication output. Notably, there is relatively low level of collaboration among institutions, which is mainly confined within countries or regions. Acknowledging this, the establishment of joint research between various research establishments or groups is crucial for future research. Breaking down the barriers to communication between nations and institutions, achieving complementary strengths, and expanding the research platform will yield significant benefits for the long-term advancement of research in this field. *Fertility and Sterility* is the journal with the highest number of published papers, and stands out as the journal with the most co-citations.

Simón, C, from the University of Valencia in Spain boasts the highest H-index and is also the author with the greatest productivity in the domain, indicating his outstanding influence and significant contributions to the domain of embryo transfer and endometrial receptivity. One of his articles published in 2020 attracted a lot of academic attention, it provides a detailed report of a 5-year multicentre randomized controlled trial aimed at contrasting the clinical efficacy of PET directed by ERA with FET and fresh embryo transfer during IVF. Although the intention-to-treat analysis did not demonstrate a significant advantage of PET at the first transfer, the per-protocol (PP) analysis indicated that PET significantly outperformed FET and fresh embryo transfer regarding pregnancy rates, implantation rates, and cumulative live birth rates. The ERA test provides a scientific basis for guiding PET and has the potential to incorporate endometrial factors into the assessment at the initial consultation for infertile patients. However, in the future, larger and more rigorous research trials need to be conducted to further verify the clinical effectiveness of PET and to assess its cost-effectiveness.

Lessey, BA, from Atrium Health Wake Forest Baptist in the USA, holds the distinction of having the greatest number of co-citations. His article titled *What exactly is endometrial receptivity?* published in *Fertility and Sterility*, advocates for a paradigm shift in assessment methods from traditional histology to modern gene expression analysis, such as ERA, and specific protein detection.^[19]^ This publication ranks second in terms of burst intensity over the past 2 decades.

As can be discerned from the foregoing results, it is manifest that the relationship between embryo transfer and endometrial receptivity has drawn increasing attention in recent years. With the advancement and breakthroughs in molecular biology and genetic testing technologies, we anticipate a broad future for this field of research.

### 4.2. The developmental trajectory and evolutionary path of the research domain

Since the birth of the world’s first test-tube baby in 1978, ART has traversed over 4 decades of history, continually advancing and refining its methods.^[20]^ The evolution of these technologies adheres to a trajectory that begins with in-depth exploration in basic research, progresses to ongoing improvements in clinical techniques, and culminates in breakthroughs and applications of advanced ART. As depicted in Figure 8B, which illustrates the timeline of keyword clustering, a transition from dark blue to light yellow of the nodes can be discerned, signifying the progression of the research domain from its inception to the most recent developments. The earlier co-cited literature keywords that have emerged can be clustered as #0, #3, #8, #11, #15, #4, #5, and #16, which can be summarized as Cryopreservation, Hormonal Regulation, and Endometrial Receptivity.

The primary developmental evolutions include advancements in cryopreservation techniques, transitioning from the initial traditional slow-freezing methods to the current vitrification technology.^[21]^ This shift has significantly enhanced the survival and implantation rates of frozen embryos. Vitrification cryopreservation avoids the formation of ice crystals through rapid cooling, thereby minimizing damage to embryonic cells.^[22]^ In the realm of hormonal regulation, research has focused on improving the endometrial environment through hormonal modulation, such as the roles of progesterone and estradiol in endometrial preparation.^[23,24]^ The application of these hormones has provided effective means for controlled ovarian stimulation, thereby enhancing the success rates of ART. Advancements in the study of endometrial receptivity encompass the development of genomic diagnostic tools based on transcriptomic signatures to ascertain the receptivity of the human endometrium. These tools are instrumental in identifying the optimal window of endometrial tolerance within the female reproductive cycle, thereby enhancing the success rates of embryo transfer.

During the developmental process, additional keywords have gradually emerged, such as clusters #2, #13, #1, #6, #12, and #14. The keywords in the literature that were co-cited clearly show that the most important areas are embryo transfer techniques, implantation failure, and maternal health. The research advancements in this group have primarily focused on enhancing the success rate of embryo transfer techniques, addressing the issue of RIF, and the impact of maternal health on fertility. From the traditional fresh embryo transfer to the development of FET and PET strategies, the aim is to enhance implantation rates and reduce the risk of implantation failure. Additionally, study on the factors behind RIF, including the thickness of the endometrium and intrauterine adhesions, has led to the exploration of novel therapeutic approaches, including the application of stem cell therapies and PRP techniques to refine the endometrial environment.^[24–26]^ Concurrently, studies are examining maternal factors such as BMI and their impact on the success rate of embryo transfer, as well as how fertility can be improved through lifestyle adjustments.^[27,28]^

The recent co-cited literature keywords, clustered as #7, #9, and #10, can be encapsulated under the themes of Advanced Therapies, Diagnostics, and Endometrial Health. The research advancements in this cluster are primarily focused on employing sophisticated therapeutic approaches and diagnostic technologies to enhance the health of the endometrium, thereby increasing the success rates of embryo transfer. Emerging treatment methods, such as the use of PRP technology,^[29,30]^ are being utilized to promote the repair of the endometrium and to augment its thickness. At the forefront of diagnostic techniques, technologies like hysteroscopically guided biopsies are improving the accuracy of intrauterine disease diagnosis,^[31,32]^ while proteomic and metabolomic assessments are being employed to evaluate the receptiveness of the endometrium.^[5,33]^ Additionally, research is delving into the physiological and traumatic repair of the endometrium,^[34]^ exploring how physical therapies and bio-barrier materials can facilitate endometrial repair and prevent intrauterine adhesions.^[35–38]^ These collective efforts aim to advance our understanding and treatment of endometrial health, ultimately contributing to the success of embryo transfer procedures.

### 4.3. Core content and hotspot analysis of the research field

Keywords and references that frequently emerge and are highly utilized over a period represent the research hotspots of that time. The statistical analysis of these keywords can track the changing trends of hotspots within the research field. Currently, the research directions in embryo transfer and endometrial receptivity are primarily focused on several major areas, including comparisons between fresh and frozen-thawed embryo transfer, RIF, and approaches to improve endometrial receptivity.

#### 4.3.1. Fresh embryo transfer versus frozen embryo transfer

With the rapid advancement of ART, IVF-ET has become a significant treatment modality for infertility. However, the levels beyond physiological hormones used throughout ovarian stimulation can result in premature endometrial maturation, reducing endometrial receptivity and consequently affecting the proportion of successful embryo implantation. Fortunately, the application of estrogen and progesterone before FET allows for thorough endometrial preparation, achieving better embryo-endometrium synchronization.^[39,40]^ Moreover, the cryopreservation process selects embryos with poorer developmental potential, consequently upgrading the quality of transferred embryos and increasing the implantation rate.^[41]^ Research has shown that in women who have polycystic ovary syndrome, the incidence of moderate to severe ovarian hyperstimulation syndrome is significantly reduced with FET compared to fresh embryo transfer.^[17]^ This reduction is possible due to the avoidance of excessive gonadotropin stimulation and elevated estrogen levels present during the fresh cycle,^[42,43]^ which can prematurely mature the endometrium and decrease its receptivity.^[44]^ Nevertheless, there exists evidence indicating that FET may potentially be linked to an augmented risk of pregnancy-induced hypertension or preeclampsia.^[17]^ These findings underscore the complex interplay between Embryo Transfer Protocols and the broader implications for maternal health during the perinatal period.^[45]^

#### 4.3.2. Repeated implantation failures

RIF is characterized by the inability to achieve successful embryo implantation following 3 or more IVF attempts with high-quality embryos.^[16,46,47]^ The etiology and pathogenesis of RIF are complex and multifactorial, typically associated with advanced maternal age, uterine factors, immunological factors, and genetic components, which can significantly impair both the psychological and physiological health of the patients.^[48]^ Research demonstrates among those patients with a record of at least one failed transfer of a top-quality blastocyst, 22.5% were diagnosed with a Displaced WOI using ERA.^[18]^ This proportion aligns with previous studies that report the contribution of endometrial factors to implantation failure, ranging from 25% to 30%. For these patients, PET and adjustment of progesterone supplementation protocols may improve the success rate of subsequent transfers of euploid embryos.^[49]^ Additionally, analyses reveal that FET may raise the possibility of preeclampsia during pregnancy. For RIF attributed to other causes, a comprehensive evaluation of the patient’s systemic condition, optimization of ART,^[50]^ anti-infective treatments, and immunotherapies can collectively enhance the success rate of implantation. Interdisciplinary collaboration and multicenter studies will provide robust support for in-depth exploration of RIF.^[51]^

#### 4.3.3. How to improve endometrial receptivity

**Figure 9.**
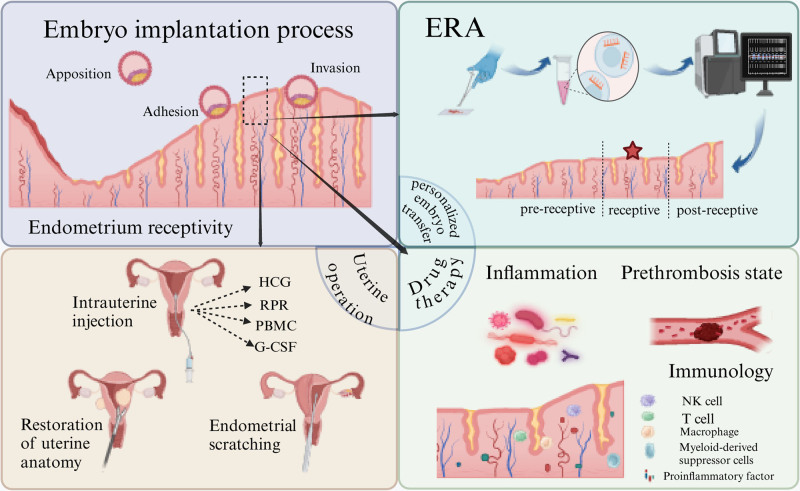
Embryo implantation goes through 3 important stages: apposition, adhesion, and invasion, and the endometrium,^[7]^ as the gateway to embryo implantation, is in a good state of tolerance, which is the key to a successful pregnancy. Endometrial receptivity can be improved by applying endometrial receptivity analysis (ERA) to assess whether it is in the ideal state to accept embryo implantation for personalized embryo transfer. Secondly, uterine operations, such as intrauterine injection of human chorionic gonadotropin (HCG), platelet-rich plasma (RPR), peripheral blood mononuclear cell (PBMC), and granulocyte colony-stimulating factor (G-CSF), can be performed to increase endometrium blood flow and improve the microenvironment of the uterine cavity. Alternatively, the uterine cavity occupancy can be removed to restore the normal anatomical shape of the uterine cavity. Endometrial scratching can also be performed to stimulate endothelial and vascular proliferation and to improve the endometrial condition for implantation. Finally, pharmacological interventions are applied. For example, oral antibiotics can be used to treat endometritis. Aspirin and low-molecular heparin can be used to improve the prethrombotic state of the sub-endometrial vessels. Oral immunosuppressants can reduce maternal rejection of the embryo. Created with BioRender.com.

Improving endometrial receptivity can be approached through various methods, as shown in Figure 9 (Confirmation of publication and licensing rights by Bio render can be found in Data S10, Supplemental Digital Content, https://links.lww.com/MD/O746): surgical treatment plays a crucial role for patients with abnormal uterine cavity morphology,^[52,53]^ such as submucosal fibroids and intrauterine adhesions.^[54–56]^ Hysteroscopy can significantly enhance endometrial receptivity by allowing a direct assessment of the uterine cavity and correcting any identified structural abnormalities. Intrauterine instillation is another therapeutic approach that involves the direct injection of medications or growth factors into the uterine cavity. Studies have shown that instillations of HCG, G-CSF, PRP, and other such agents can markedly improve endometrial receptivity, thereby increasing the incidences of embryo implantation and clinical pregnancy.^[57–60]^ Pharmacological treatment is also a common method used to enhance endometrial receptivity. Medications such as estrogen, low-dose aspirin, low-molecular heparin, and pentoxifylline combined with vitamin E primarily act by increasing endometrial thickness and uterine artery blood flow, which subsequently enhances endometrial tolerance.^[61,62]^ Endometrial scraping is a method aimed at enhancing endometrial receptivity through induced endometrial injury.^[63,64]^ The potential mechanisms include the secretion of pro-inflammatory substances conducive to implantation (such as interleukins, growth factors, etc), recruitment of macrophages, and induction of endometrial decidualization.^[32]^ Chronic endometritis leads to an increase in inflammatory mediators, activation of immune cells, a decrease in endometrial receptivity, and may elicit a rejection response to the embryo, affecting implantation.^[65]^ Treatment primarily involves oral antibiotic therapy, with therapeutic efficacy assessed post-treatment through hysteroscopic examination combined with biopsy.^[32]^ Reports indicate that in patients with RIF, group witnessed a notable surge in both pregnancy and live birth rates where chronic endometritis was cured.^[51,66–68]^ In addition, traditional Chinese medicine therapies, including herbs that activate blood circulation and acupuncture,^[69]^ are thought to improve the receptivity of the endometrium.^[70–72]^ These methods work by regulating uterine blood flow and promoting endometrial growth, thus enhancing the uterine lining’s receptivity to embryo implantation.

### 4.4. Frontier exploration and trend forecasting: applying ERA personalized migration

Embryo implantation represents a complex biological process characterized by a dynamic dialogue between the embryo and the endometrium. This intricate interaction is crucial for the achievement of a viable pregnancy and remains an object of intense research within the domain of reproductive biology,^[73]^ with the receptivity of the endometrium playing a crucial role in this interaction.^[74]^ Standards for endometrial receptivity can be broadly categorized into several classes: ultrasonographic assessment, endometrial volume, endometrial pattern, Doppler signals, endometrial wave-like activity, endometrial biopsy, endometrial fluid aspiration, and hysteroscopic examination.^[75]^ The traditional view of endometrial receptivity as a binary variable (present or absent) may be overly simplistic. In fact, endometrial receptivity may be a continuous variable that reflects the molecular changes occurring in the endometrium affected by ovulation and progesterone exposure. It is necessary to rethink the traditional of the description window as a dynamic process in which endometrial receptivity gradually changes.^[76]^ Historically, endometrial receptivity has been assessed through histological examination of endometrial tissue, a method that is fraught with limitations, including lack of precision and significant subjectivity. With the advent of molecular biology techniques, a variety of novel assessment methods have emerged, such as integrin detection,^[77]^ and ERA.^[78]^ These approaches offer a more objective and accurate means of evaluating endometrial receptivity, potentially allowing for a more nuanced understanding of the receptivity continuum rather than a binary classification. Researchers have developed a genomic tool known as ERA over the past decade, based on comprehensive transcriptomic analyses of the human endometrium. This tool is grounded in a customized microarray and integrates specially trained bioinformatics-based predictive computer algorithms for the identification of the timing of the WOI within the endometrium.^[79–81]^ The ERA has been shown to exhibit greater accuracy and consistency than histological (Noyes) dating in identifying the personalized WOI days,^[82]^ consequently giving rise to the new clinical concept of PET on the optimal day of endometrial receptivity.^[18,83]^ This objective and reproducible molecular detection method analyzes the manifestation of over 200 genes in endometrial tissue to accurately and steadily recognize the tolerance condition of the endometrium, thus providing guidance for PET Studies have shown that pregnancy and implantation rates in the PET group are markedly above in the FET group at both the initial and cumulative levels, and are close to or significantly higher than those in the fresh embryo transfer group. Personalized medicine, while in its early stages in the field of reproductive medicine, has achieved personalized diagnosis and precision treatment of endometrial factors through ERA testing, offering a new avenue to enhance the performance metrics of IVF.^[3]^ However, continued research is needed to refine and optimize this technology to realize its full potential.^[84]^

### 4.5. Advantages and constraints

To our knowledge, this is the first bibliometric report on embryo transfer and endometrial receptivity. In our research, we employed a triad of bibliometric methodologies, CiteSpace, VOSviewer, and R-bibliometrics, to synthesize the analysis, thereby yielding a more holistic understanding of the technological domain in question. Nonetheless, this research is accompanied by certain constraints. Firstly, due to the challenges in consolidating data from disparate databases using these bibliometric software tools, we confined our analysis to records within the WoSCC database, possibly omitting pertinent research of other high-quality databases. Then, by focusing solely on English-language literature, we may have underestimated the contributions from non-English-speaking countries. Third, the ongoing updates to the WoSCC database could lead to an underestimation of the influence exerted by recently published, high-caliber papers, given that they might not have amassed a substantial citation count as of yet.

## 5. Conclusion

In summary, the field of embryo transfer and endometrial receptivity has witnessed continuous growth and breakthroughs over the past 2 decades. Our comprehensive analysis of the scientific literature in this domain encompassed a variety of perspectives, including global research trends, prominent journals, and key contributors. More importantly, we have provided a detailed overview of the current main research directions and focal points, along with the most anticipated explore frontiers and hotspots for the future. Collectively, our bibliometric study offers valuable insights for subsequent investigators to better comprehend the foundational knowledge landscape, identify potential collaboration opportunities with other research groups, pinpoint current research hotspots, and grasp forthcoming research frontiers.

## Author contributions

**Data curation:** Chen Xu, Chuanhui Zhang.

**Formal analysis:** Chen Xu.

**Investigation:** Chen Xu, Chuanhui Zhang.

**Methodology:** Chen Xu.

**Project administration:** Chen Xu.

**Resources:** Chen Xu.

**Supervision:** Chen Xu.

**Visualization:** Shu Xu, Jingli Ma, Lingling Ran.

**Writing – original draft:** Chen Xu.

**Writing – review & editing:** Chen Xu.

## Supplementary Material


